# An Update on Molecular Pathways Regulating Vasculogenic Mimicry in Human Osteosarcoma and Their Role in Canine Oncology

**DOI:** 10.3389/fvets.2021.722432

**Published:** 2021-09-23

**Authors:** Marcella Massimini, Mariarita Romanucci, Raffaella De Maria, Leonardo Della Salda

**Affiliations:** ^1^Faculty of Veterinary Medicine, University of Teramo, Teramo, Italy; ^2^Faculty of Veterinary Medicine, University of Turin, Turin, Italy

**Keywords:** comparative oncology, dog, osteosarcoma, vasculogenic mimicry, molecular pathways

## Abstract

Canine tumors are valuable comparative models for human counterparts, especially to explore novel biomarkers and to understand pathways and processes involved in metastasis. Vasculogenic mimicry (VM) is a unique property of malignant cancer cells which promote metastasis. Thus, it represents an opportunity to investigate both the molecular mechanisms and the therapeutic targets of a crucial phenotypic malignant switch. Although this biological process has been largely investigated in different human cancer types, including osteosarcoma, it is still largely unknown in veterinary pathology, where it has been mainly explored in canine mammary tumors. The presence of VM in human osteosarcoma is associated with poor clinical outcome, reduced patient survival, and increased risk of metastasis and it shares the main pathways involved in other type of human tumors. This review illustrates the main findings concerning the VM process in human osteosarcoma, search for the related current knowledge in canine pathology and oncology, and potential involvement of multiple pathways in VM formation, in order to provide a basis for future investigations on VM in canine tumors.

## Introduction

Vasculogenic mimicry (VM) is a unique ability of malignant cancer cells to create their own fluid-conducting microvascular channels without the involvement of endothelial cells. It was firstly described in human uveal melanomas as periodic acid–Schiff (PAS)-positive microvascular channel networks (1). Since then, VM has been observed in a variety of human malignant tumors, including osteosarcoma (OSA), glioblastoma and gallbladder, ovarian, prostate, lung, gastric, hepatocellular, and breast cancer (2, 3). In addition, the presence of VM has been associated with high tumor grade, invasion, metastasis, and poor prognosis in cancer patients (4, 5). Thus, VM has emerged as a potential target for anti-tumor therapy (2, 3, 6).

In veterinary pathology, the VM process has been demonstrated in canine inflammatory mammary carcinomas and in a palpebral melanocytoma (7, 8). Rasotto et al. explored the presence of VM in primary canine mammary tumors, revealing no relation with lymphatic infiltration (9). As well, primary cell lines from canine mammary tumors, showing ability to form VM *in vitro* and *in vivo*, have been recently established and characterized (10–12). Moreover, canine inflammatory mammary carcinomas were analyzed for the presence of VM by transmission and scanning electron microscopy (13). In addition, as far as canine OSA is concerned, the presence of vessel-like structures in a long-term canine D17 OSA cell cultured on type I collagen has been recently described (14). As well, treatment with the heat shock protein 90 (Hsp90) inhibitor 17-N-allylamino-17-demethoxygeldanamycin (17-AAG) inhibited the migration of D17 OSA cells, also decreasing VM markers *in vitro* and inducing a reduction of hypoxia-inducible factor 1α (HIF1α) transcript and protein expression (14). Notwithstanding this, information regarding VM formation, molecular features, and prognostic implications in canine oncology is still limited.

Since VM has been known from a relatively short time, the molecular mechanisms involved in this process remain largely unknown. Aggressive tumor cells capable of VM display a varied gene profile which includes that of fibroblasts and epithelial and endothelial cells (15). Hypoxia, epithelial-mesenchymal transition (EMT) and in particular epithelial-endothelial transition (EET), response to extracellular matrix (ECM), and the presence of cancer stem cells (CSCs) are considered the key regulators of VM (16, 17). Various signaling pathways, promoting tumor migration and invasion, have been reported to participate in VM formation, including those involved in vasculogenesis such as vascular endothelial (VE)-cadherin, vascular endothelial growth factor (VEGF)/VEGF receptor (VEGFR) and platelet-derived growth factor (PDGF)/PDGF receptor (PDGFR) axis, and HIF1α (3). VM progression is also mediated by pathways involved in ECM adhesion and cell migration, such as focal adhesion kinase (FAK) and migration inducting gene 7 (*Mig7*) encoding for breast cancer anti-estrogen resistance protein 3 (BCARP 3), matrix metalloproteinases (MMPs), integrins and erythropoietin-producing hepatocellular receptorA2 (EphA2), as well as multiple signaling pathways including mechanistic target of rapamycin (mTOR) and Rho-associated coiled-coil kinase (RhoA/ROCK) (3). Finally, increasing evidence showed that VM can be affected by microRNA (miRNA), long non-coding RNA (lncRNA), and circular RNA (circRNA) (18).

**Figure 1:**
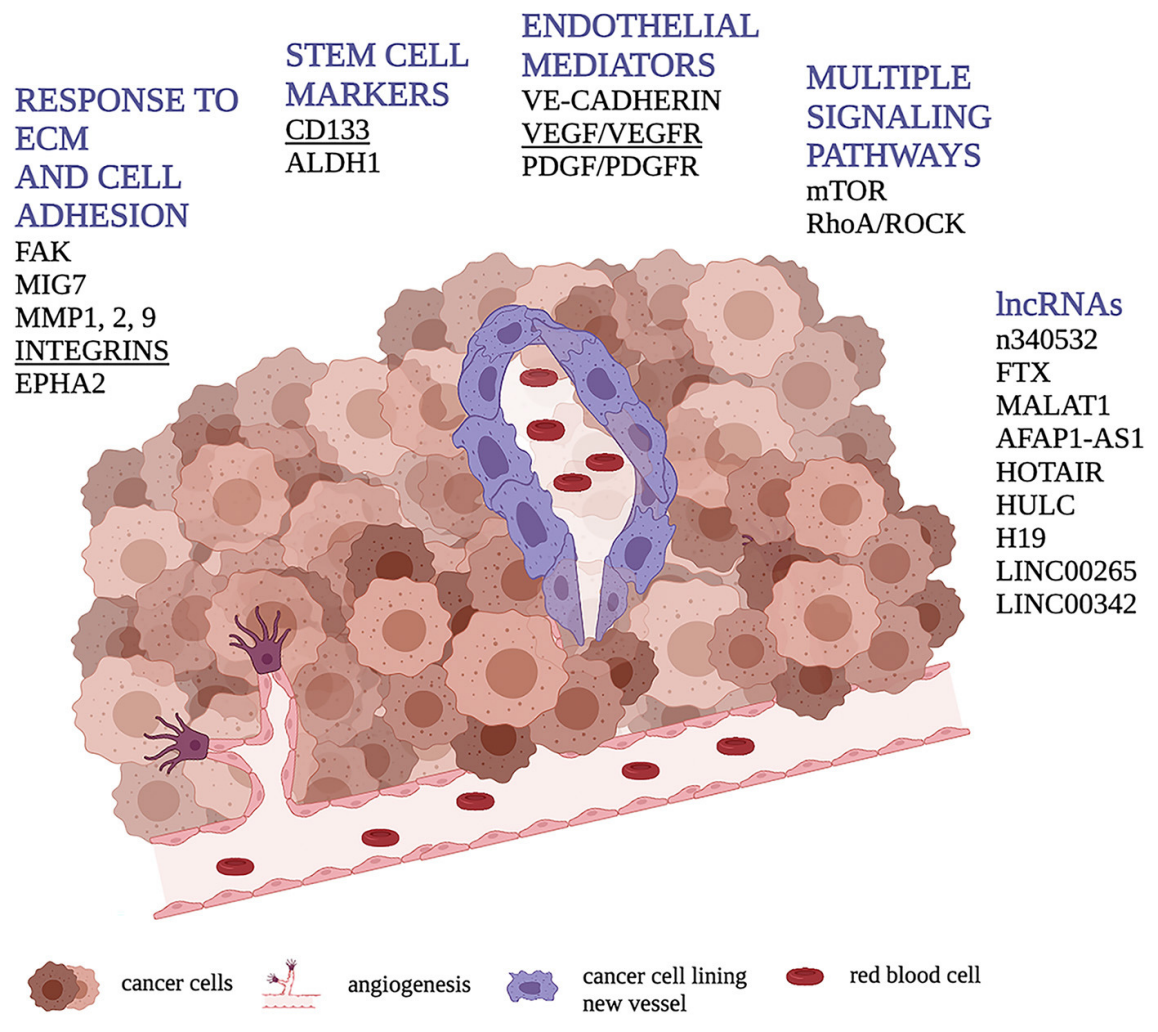
Schematic representation of VM through cancer cells (in purple) forming a vessel containing red blood cells. Figure shows the main molecular pathways involved in the VM process in human osteosarcoma highlighting, in underlined bold, those found to be related with VM presence or tubular/vessel-like formation *in vitro* in dog.

**Figure 2:**
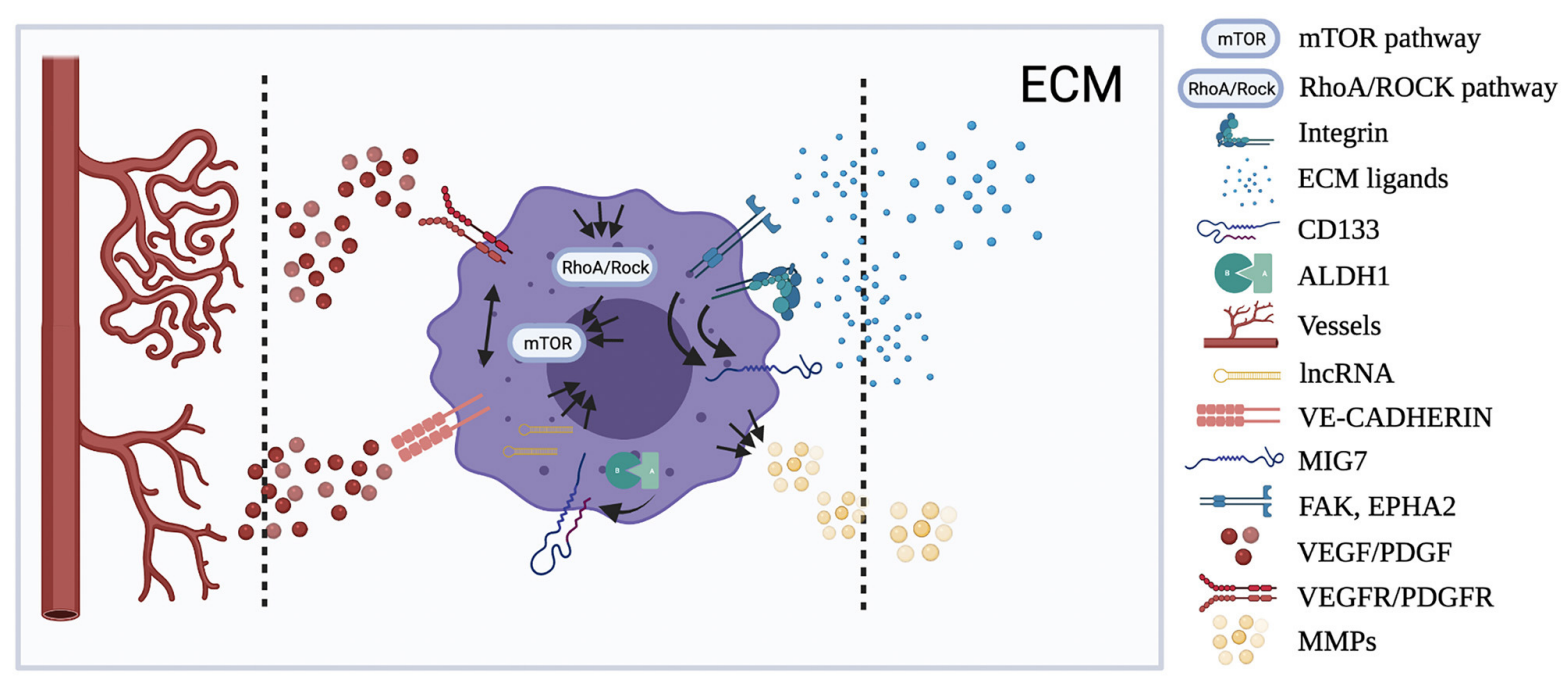
Localization of the principal molecular pathways involved in VM. Figure shows the cellular and tumor microenvironmental distribution of the human OSA pathways resumed in the review showing, when known, the possible interactions (black arrow) between them. Multiple arrows show multiple interactions between pathways.

Thus, the aim of this review is to illustrate the main findings concerning the VM process in human OSA (Figures 1, 2), as well as the current knowledge on the molecular pathways potentially involved in VM formation in canine pathology and oncology (Supplementary Table 1), in order to provide a basis for establishing further investigations on VM in canine tumors in the future.

## CSCs Markers: CD133 and Aldehyde Dehydrogenase 1 (ALDH1)

CSCs represent an important feature of VM progression for their ability to differentiate in endothelial cells forming new microvessels (19). Stemness and differentiation potential of CSCs are enhanced under hypoxic microenvironments, through hypoxia-induced EET and ECM remodeling, thus determining the formation of the specific features of VM (17). Bao et al. (30) described a positive correlation between CD133 expression and presence of VM in OSA, which was, in turn, positively associated with ALDH1 expression. CD133, also called prominin-1, is a common biomarker of CSCs, which encodes a 120-kDa five-transmembrane domain glycoprotein. Its dysregulation has been considered as a CSC biomarker in various human cancers including OSA (20, 21), and it is correlated with VM, presence of metastasis, and poor prognosis in different tumors (21, 22). Little is still known about the mechanisms used by CSCs for promoting angiogenesis and VM (23). In this respect, the ability of CD133 to activate the Wingless-related integration site (Wnt) signaling pathway, thus increasing the expression of VEGFα and interleukin-8 (IL-8) (24), and its mechanistic link with cell motility (25), may be involved in the VM process.

ALDH1 is another common biomarker of dysregulated CSCs in a variety of human cancers (26, 27), the inhibition of which could represent a target in OSA therapy (28, 29). In the study of Bao et al. (30) multivariate analysis data showed that the expression of CD133, ALDH1, and VM; grade of differentiation; recurrence; as well as Enneking stages were independent prognostic factors for OSA patients. Despite the identified correlation with prognosis, the presence of CSC markers lining VM-dependent vessels has not been demonstrated in OSA tissues, even though CD133+ stem-like cell accumulation has been observed in the melanoma perivascular niche (30, 31).

### CD133 and ALDH1 as Canine CSC Markers and Their Expression in Madin–Darby Canine Kidney Cells

The general structure of prominin-1, including its membrane topology, has been conserved throughout the animal kingdom (32). Non-tumor canine cells, in particular MDCK cells, have been widely used for understanding the mechanisms on the basis of cell motility. In fact, considering that prominin-1 is associated with plasma membrane protrusions, the overexpression of *Prom1* gene increased the number of MDCK microvilli, while the overexpression of a dominant-negative mutant variant significantly decreased ciliary length (25). The involvement of CD133 in cell motility was also demonstrated by Liu et al., showing the ability of isolated canine CD133+ epithelial cells to form a tubular-like structure when cultured on Matrigel (33). Likewise, endothelial progenitor cells isolated from canine bone marrow CD133+ are capable of forming a capillary structure on Matrigel after 24 h of culture and can be transplanted in ischemic injured tissues to enable neovascularization (34, 35).

In cancer, CD133 staining, together with functional properties including ALDH enzyme activity and spheroids formation *in vitro*, is commonly used to characterize potential CSCs in canine OSA and others types of spontaneous canine cancer, not only those deriving from a hematopoietic lineage (36–40). Immunohistochemical investigations revealed that CD133 was expressed in all grades of OSA, glioma, melanoma, hepatocellular carcinoma, B-cell lymphoma, and granular cell tumor, with a higher proportion of positive cells in high-grade tumors (41–45).

Although a direct association between CD133 and VM has not been investigated in canine tumors, CD133+ cancer cells showed different features linked to VM both *in vivo* and *in vitro*. Highly invasive and tumorigenic canine insulinoma CSC-like cells and canine prostate cancer cellsCD133+ showed an invasive and tumorigenic phenotype *in vivo*, similar to hepatocellular carcinoma and lung adenocarcinoma cell lines that were also capable of forming spheroids in culture (46–49). CD133+ hemangiosarcoma cell lines cultured under normal and sphere-forming conditions generated three distinct tumor subtypes *in vitro*, associated with angiogenesis, inflammation, and adipogenesis (50). CD133+ canine cell lines derived from OSA, melanoma, transitional cell carcinoma, and lung adenocarcinoma resulted to be significantly resistant against X-ray irradiation (38). Gatti et al. observed that canine OSA primary cultures containing CD133+ CSCs exhibited distinctive sensitivity to anticancer agents (51), as well as spheroids derived from canine mammary gland adenocarcinoma (52).

As far as ALDH1 is concerned, despite ALDH enzymatic activity being also considered a cancer marker in canine samples (37, 53, 54), to the best of our knowledge, its protein and gene expression has not been directly investigated in canine tumors, as well as in normal tissues or in other canine pathological conditions (55–57).

## Endothelial Mediators

VM takes place independently of angiogenesis or endothelial cell proliferation, although it is often associated with endothelial marker expression (15). Gene expression analysis showed that aggressive tumor cells capable of VM display a diversified gene profile, expressing genes from multiple cell types including those of endothelial cells (58). In fact, the concept of “embryonic-like and vascular phenotype in the absence of endothelial markers,” referred to as the first histological definition of VM (1), is controversial. In this respect, it has been demonstrated that primary and established sarcoma cell lines, after prolonged stimulation with post-surgery fluids from a cohort of patients affected by giant cell tumors of bone, transdifferentiated into VE-Cadherin+ and CD31+tubular-like structures (59). For this reason, the term “endothelial mediators” (and not endothelial markers) is preferred, to avoid controversy concerning the attribution of specific endothelial markers to highly aggressive cells that undergo VM. In several tumors, especially melanoma, an important group of endothelial mediators has been found in association with VM, including VE-cadherin (60–62) and VEGFR1 (63).

In MG63 OSA cells, the inhibition of *Cdh5* gene encoding for VE-cadherin with small interfering RNA (siRNA) reduced the ability of cells to form endothelial-like networks when cultured on type I collagen or Matrigel (64), and the same phenomenon has been observed in silencing the *Vegf* gene (65). In fact, autocrine VEGF/VEGFR1 signaling, associated with increased tumor growth and tumor vascularity, may possibly confer the capacity to develop vasculogenic properties to OSA cells (66). In recent studies, differentially expressed genes (DEGs) were investigated between different OSA cells cultured on Matrigel for profiling the molecular patterns involved in VM phenotypes. Results from these studies showed that the endothelial mediators PDGFRα and PDGFRβ were correlated with malignancy and tubular-like structure formation *in vitro* (67, 68).

### VE-Cadherin as Regulator of EMT and Vascular Integrity in Canine Pathology

VE-cadherin is an endothelial cell-specific cadherin that functions to stabilize cell structure because of its involvement in calcium-dependent intercellular adhesion (69). In dog, it has not been linked with VM, nor investigated in MDCK cells, although its role in EMT, a process closely related to VM and vascular integrity, has been explored (70, 71). In fact, VE-cadherin gene expression and immunohistochemical staining was evaluated in canine myxomatous mitral valve disease to investigate the role of EMT in chronic valvulopathies, showing a significant *cdh5* gene dysregulation (72).

In cancer, VE-cadherin protein expression was observed at intercellular junctions in both normal canine tissue-derived cells (NECs) and in canine tumor-derived cells (TECs), isolated from thyroid carcinoma and perianal gland epithelioma. The observed zigzag pattern in TECs, with respect to the linear in NECs, may be indicative of VE-cadherin dysfunction and increased vascular permeability, probably dependent on the high concentration of VEGF in the tumor microenvironment *in vivo*. In fact, an abnormal VE-cadherin expression pattern was observed in 100% confluent NECs, following culture in a tumor-conditioned medium containing excessive VEGF (73). Moreover, this study showed that Combretastatin A-4 phosphate (CA4P) has selective effects on TEC morphology and NECs in tumor culture conditions, also disrupting vasculature in canine OSA xenografted into mice (74). Furthermore, genome-wide methylation analysis performed in canine mammary tumors showed a significant hypermethylation at the PAX5 (paired box protein 5) motifs in the intron regions of *cdh5* gene and a consequent gene down-regulation (75).

### VEGF/VEGFR Axis in Relation to VM in Canine Osteosarcoma and Mammary Tumors

As far as VEGF/VEGFR axis in veterinary oncology is concerned, VEGF family members were identified in several canine cancers (76), as well as OSA tissue, serum, and cultured cells (77–79). A relation between VM and VERGFR was found in D17 canine OSA cells cultured on type I collagen where malignant cancer cells with endothelial morphology express VEGFR1 (14). Correlation between VM and VEGF axis has been firstly investigated in dogs with mammary tumors (7). VEGFα, VEGFγ, and VEGFR3 were expressed in spontaneous canine mammary tumor and xenograft models (80), showing increased expression in the inflammatory mammary carcinoma (IMC) model compared to non-IMC and mammary OSA (80, 81). VM has been shown to occur more frequently in IC compared with other types of canine mammary tumors (7). Furthermore, overexpression of VEGFα, VEGFγ, and VEGFR3 was observed in canine malignant non-IMC, and it was correlated with cyclooxygenase 2 (COX2) immunoexpression, which is particularly related to VM progression (82, 83).

Another indirect relation to VM can be found in the study of Cam et al. in which VEGFα expression in different OSA cell lines and its correlation with ΔNp63 and cell migration on Matrigel was described, demonstrating that ΔNp63 exerts its angiogenesis and invasion property through VEGFα (84). It has been also demonstrated that VEGFα is the direct target of miR34a, which is less expressed in OSA cell lines with respect to normal osteoblast; OSA cells that have been induced to overexpressing miR34a show decreased motility and invasion ability on Matrigel and increased levels of VEGFα (85). On the other hand, a correlation between VEGFα transcript and chemical hypoxia was not observed (77).

As far as OSA *ex vivo* samples are concerned, literature data regarding VEGF axis and VM are lacking. Moreover, no correlation was observed between VEGF expression and clinicopathological parameters or hypoxia markers, which are often related to VM (77). On the contrary, its serum concentration has been previously correlated with poor prognosis in canine OSA (86). Increased levels of VEGF in serum or in cell supernatant were also observed after treatment of canine OSA with tyrosine kinase inhibitors such as Toceranib, Erlotinib, and Masitinib mesylate, probably due to a mechanism of feedback response to VEGFR2 inhibition (87–90).

### PDGF/PDGFR Axis in Osteosarcoma and Other Canine Tumors

An extensive knowledge concerning PDGF/PDGFR axis is available in veterinary literature. This molecular axis has been investigated as endothelial marker (91), in wound healing (92), spontaneous canine astrocytoma (93), fibrosarcoma (94), squamous cell carcinoma (95), lymphoma (96), prostate cancer (97), hemangioma and hemangiosarcoma (98), melanoma (99), mast cell tumors (100), hepatocellular carcinoma (101), mammary tumors (102), and nervous system tumors (103, 104).

PDGFs and PDGFRs were also found to be coexpressed and overexpressed in canine OSA, suggesting an autocrine and/or paracrine loop. In particular, in the study of Maniscalco et al. (105) all evaluated canine OSA cell lines overexpressed PDGFRα, while 6/7 overexpressed PDGFRβ, when compared to a normal osteoblastic cell line (106). The involvement of an autocrine loop of PDGF signaling pathway in the pathogenesis of canine OSA was confirmed in other studies, showing the overexpression of *cis*, the coding gene of PDGFRβ, in a OSA cell line (CO8), and the ability of its supernatant to induce tyrosine phosphorylation and therefore the activation of the PDGFRα and PDGFRβ on murine 3T3 cells (107, 108). Meyer et al. demonstrated that, in addition to tumor cells, giant cells and osteoblasts in canine OSA were positive for PDGFBB immunostaining, composed of two subunits β, also showing the detection of its mRNA in all study cases (109). Finally, the dysregulation of the expression levels of PDGFRβ in canine OSA has been attributed to the strong demethylation of CpG sites within the promoter (110).

No evidence currently exists concerning a relationship between VM and PDGF/PDGFR axis in canine oncology. Furthermore, no significant correlation was observed between the expression of these molecules and survival or histological grading in canine OSA (105). Despite this, the significant relation of this axis with malignant features of canine OSA has been observed both *in vivo* and *in vitro* (111). In fact, treating OSA cells with Toracenib, a potent inhibitor of PDGFRs, has been shown to induce a decrease in cell growth, migration, motility, and colony formation, as well as a significant blunting of tumor growth and proliferation index in an orthotopic xenograft model (111). These findings suggest that PDGF/PDGFR axis can represent a target therapy more than a diagnostic tool. With the coming of new technologies linked to miRNA, miR34a was tested on OSA cell lines and xenograft mouse models, showing PDGFRα reduction, together with decrease in cell proliferation and migration *in vitro* and tumor growth *in vivo* (112).

## Response to ECM Environment and Cell Adhesion

Among the myriad of microenvironmental factors affecting cancer cell resistance, cell adhesion to the ECM has been recently identified as a key determinant (113). FAK is a non-receptor tyrosine kinase that mediates signaling events downstream of integrin engagement of the ECM, regulating cell survival, proliferation, and migration and supporting neovascularization and maintenance of CSCs (114). FAK is expressed in different cancer types, where it is involved in the progression of tumor aggressiveness. Small molecule FAK inhibitors in clinical phase trials demonstrated to be effective in cancer by inducing tumor cell apoptosis in addition to reducing metastasis and angiogenesis (115). Association between FAK and VM or invasive behavior has been observed in different cancer types, including OSA. Ren et al. showed FAK staining in the cytoplasm of OSA tissue cells with high intensity around VM vessels (116). Similarly, *Mig7* gene was expressed in the cytoplasm with higher percentage of positivity in the VM with respect to non-VM group, suggesting an association between Mig7expression and VM formation and identifying in VM a prognostic marker of OSA (116). *Mig7* protein is enriched in embryonic cytotrophoblast cells during placental development and in more than 80% of tumors compared to normal tissue samples and blood from normal subjects (117). It was found to colocalize with VE-cadherin in cells lining VM structures in a lymph node metastasis (118) and to initiate a signaling cascade that results in tumor VM (119, 120). Moreover, *Mig7* knockdown inhibited tubular-like vessel formation and invasion of MG63 and 143B OSA cells cultured on Matrigel, as well as growth and metastasis of OSA cells in a mouse model (121). *Parispolyphylla*, from traditional Chinese medicine, inhibited cell migration, invasion, and VM formation *in vitro* and *in vivo* by reducing expression of FAK, Mig7, MMP2 (gelatinase A), and MMP9 (gelatinase B) (122). MMP1 (interstitial collagenase) also resulted to be the first upregulated gene among the DEGs of the abovementioned studies performed on OSA cells cultured on Matrigel (67, 68).

Among the plethora of membrane proteins interacting with the ECM, integrin-α2 (ITGA2) has acquired an important role for its involvement in tumor cell proliferation, invasion, metastasis, and angiogenesis. In fact, its abnormal expression correlates with unfavorable prognosis in multiple types of cancer (123). *Itga2* gene overexpression has been reported to be related to increased OSA metastasis and invasion (124) and was upregulated in malignant OSA cells *in vitro* (68). In the study of Yao et al., gene signal transduction networks (Signal-net) were performed to identify the key genes involved in VM formation in OSA and the top-ranked ones resulted to be *Itga2*, integrin subunit alpha 1 (*Itga1*) and integrin subunit alpha 6 (*Itga6*) together with protein kinase cAMP-activated catalytic subunit beta (*Prkacb*), actinin alpha 1(*Actn1*), actinin alpha 4 (*Actn4*), phospholipase C beta 4 (*Plcb4*), gap junction protein alpha 1(*Gja1*), and the already mentioned gene encoding for PDGFRβ and PDGFα. Finally, this study demonstrated that *Itga1* knockdown inhibited VM formation by 143B cells *in vitro* and *in vivo* (68).

In addition, the tyrosine kinase EphA2, which belongs to the family of Eph tyrosine kinase receptors, is highly expressed in tumors, while it has been found at relatively low levels in most normal adult tissues, indicating its potential application in cancer treatment (125). Recent evidence suggests that VM occurrence is positively correlated with high expression of EphA2 and that its gene silencing inhibits VM formation (126). Interesting is also the correlation with Epstein–Barr virus (EBV) infection that stimulates plasticity in epithelial cells to express an endothelial phenotype (127). As well, Zhang et al. demonstrated that *Epha2* gene silencing inhibited VM formation in MG63 OSA cells (128).

### FAK Protein in Canine Tumor Progression

Interactions between tumor cells and tumor microenvironment are considered critical in carcinogenesis, tumor invasion, and metastasis (129). The involvement of adhesion proteins in canine OSA has been demonstrated through an expression profiling comparison between dogs with disease-free intervals (DFI) of <100 and >300 days (130).

The study of Brachelente et al. exploring the differential expression between melanomas and melanocytomas, identified differentially expressed gene clusters including nine genes belonging to the focal adhesion family (129). As far as FAK protein in humans is concerned, it is well-established that FAK serves as a scaffold for multiple protein signaling complexes, and its scaffolding function is very important for tumor progression (131). In canine oncology, interesting results were shown by Rizzo et al., demonstrating that the treatment of highly invasive D17 cells and other two OSA cell lines with Sulforaphane significantly decreased the phosphorylated state of FAK, also diminishing the invasion ability of cells cultured on Matrigel (132). These findings indirectly suggest a correlation between FAK activity and VM, considering that the inhibition of D17 OSA cell invasiveness corresponds to a decrease of VM features *in vitro* (14). Moreover, inhibition of FAK phosphorylation improved migration of canine hemangiosarcoma cells (133). FAK-mediated signaling was induced by numerous microenvironmental inputs and plays a central role in tumor-associated EMT and epithelial cells extrusion, migration, and response to the transforming growth factorβ (TGFβ) and the hepatocyte growth factor (HGF), as often demonstrated on MDCK cells (134–140). The use of these cells has also allowed understanding the involvement of FAK in the EMT induced by latent membrane protein 1 (LMP1) of EBV (141). Finally, the FAK inhibitor Masitinib mesylate (AB1010) has been the first anticancer therapy approved in veterinary medicine for the treatment of unresectable canine mast cell tumors (142).

### MMPs in Canine Tumors

In veterinary literature, current knowledge on the activity and function of proteases and stroma and their relationship with canine cancer malignancy is still limited (143), despite the fact that MMPs have been widely explored in several human cancers and are strictly related to the VM process (144, 145). Inhibition of extracellular proteolysis, in particular of collagenases MMP1, MMP2, and MMP9, is recognized as a valid approach to canine cancer therapy including OSA (146). In fact, Doxycycline at doses >5 μg/ml significantly decreased OSA cell proliferation and MMP1 activity *in vitro* (147).

*Mmp1* is the most significantly downregulated gene in Hsp70 knockdown canine OSA cells, and increased expression of *mmp2* and *mmp9* was linked to increased invasive capability in canine OSA (78, 148, 149).

Furthermore, MMP2 and MMP9 enzyme activity was found by means of zymography in three high malignant OSA cell lines (150).

The association between collagenase expression and activity and histological grade has also been demonstrated in canine mast cell tumor and lymphoma, together with VEGF dysregulation (151, 152), in mammary tumors, in relation to E-cadherin (153, 154), and in chondrosarcoma (153, 155–160). No differences in MMP9 expression were observed between IMC and non-IMC, although its expression was associated with higher nuclear grade in IMC tumors (161). As well, MMP2 and MMP9 dysregulation was found in canine oronasal tumors, hemangiosarcomas, and meningiomas, not always in association with malignant morphological patterns (143).

### Integrin Signaling in MDCK Cells and Canine Cancers

Integrin subunits may combine each other to affect the characteristics of cancer cells and the progression of tumors, both binding with proteins that directly regulate the actin cytoskeleton of cells and by phosphorylating the relative kinases, including FAKs (162). It is well-known that integrin complexes bind ECM components to promote cell adhesion and invasion, also mediating tissue tropism (163, 164). MDCK cells were used to demonstrate that α2β1 integrin mediates adhesion to types I and IV collagen in an Mg^2+^-dependent manner, thus improving cell survival, EMT, cell spreading, and brunching morphogenesis. Furthermore, overexpression of Galectin8, which activates selective β1-integrins involved in EMT, promotes oncogenic-like transformation of MDCK cells (134, 154, 165, 166).

In veterinary oncology, a deregulation of integrin pathway, together with Wnt and chemokine/cytokine signaling, has been found in relation to short survival in canine OSA (167). The expressions of β1 integrin and α5β1 complex were immunohistochemically evaluated in a series of normal, dysplastic, and neoplastic canine mammary glands, and in lymph node metastases (168, 169), while β2 integrin was found in canine cutaneous histiocytoma (170). Finally, canine hemangiosarcoma cell lines expressing several endothelial mediators including VEGF and αvβ3 integrin recapitulate features of mitotically activated endothelia and stimulate robust angiogenic responses in mice, forming tumor masses composed of aberrant vascular channels. Furthermore, they showed anchorage-independent growth and were motile and invasive, forming vessel-like structures when cultured on a basement membrane matrix (171, 172).

### EphA2 Inhibition in Canine Tumor Therapy and Its Mechanisms of Action

Targeting EphA2 represents an important goal in the development of recent anti-cancer drugs also in veterinary medicine, as shown by the attempt to evaluate the mechanism of Desanitib in the treatment of canine histiocytic sarcoma and the development of a cytotoxic compound that targets EphA2, EphA3, EphAB2, and interleukin 31 receptor A2 (IL31RA2) in canine high-grade gliomas (173, 174). The inhibition of EphA2 and IL31RA activity reduced up to 94% of tumor volume in 50% of dogs in the cohort (175). Furthermore, dogs were used to test the performance of a nanotherapeutic encapsulating a hydrolytically sensitive Docetaxel prodrug and conjugated to an antibody specific for EphA2, demonstrating an improvement in tumor penetration and antitumor activity (174). In *ex vivo* specimens, EphA2 resulted to be highly overexpressed in neoplastic cells of canine appendicular OSA, together with EphA3 (176). *In vitro, ephA2* expression was increased by up to 60-fold in canine prostate carcinoma lines derived from lung or bone metastases (177). MDCK cells were used to demonstrate the role of EphA2 in the epithelial morphogenesis in 3D culture and in the apical extrusion of transformed epithelial cells as a protective event. MDCK cells were also used to investigate EphA2 role in the decreased integration of claudin4 into sites of cell–cell contact as tumorigenic trigger and in the anoikis resistance process (178–181).

## mTOR and RhoA/ROCK Pathways

DEP domain-containing mTOR-interacting protein (DEPTOR) is an important modulator of mTOR, a kinase at the center of two important protein complexes named mTORC1 and mTORC2 (182). DEPTOR is able to interact with mTOR, thus inhibiting its kinase activity. It is involved in several molecular pathways controlling cellular homeostasis and it can behave either as an oncogene or oncosuppressor, depending on the cell or tissue type (183). It has been demonstrated that DEPTOR knockdown significantly decreased the number of tube-like structures and the invasion ability of the methylnitronitrosoguanidine transformed human OSA cells (MNNG/HOS) (184).

RhoA/ROCK pathway is a versatile regulator of multiple cellular processes, and it is dysregulated in several cancers. Recently, ROCK has attracted attention for its crucial role in angiogenesis, in regulating permeability, migration, proliferation, and tubulogenesis of endothelial cells (185). RhoA/ROCK stabilizes HIF1α during hypoxia inducing VM in hepatocellular carcinoma (186). Moreover, RhoA/ROCK expression was found to be higher in human OSA tissues and in the human OSA cell line U2OS with respect to control. Inhibition of RhoA/ROCK signaling pathway by the pharmacological inhibitor Fasudil reduced vascular-like channels in U2OS and melanoma cells cultured on Matrigel, decreasing cell plasticity and motility, both of which play key roles in VM formation (187, 188).

### Role of mTOR Pathway in Canine MDCK Cells and Cancers

mTOR pathway belongs to the series of conserved pathways that impact upon longevity and aging-related diseases such as cancer (189). Phosphatidyl inositol 3-kinase (PI3K)-AKT-mTOR was identified as one of the most relevant pathways involved in OSA progression both in humans and canines (190). The screening of protein kinase inhibitor compounds, particularly against PI3K-AKT-mTOR activity, represents an important topic of canine OSA therapy (191–193). Although the effect of the aberrant PI3K-AKT-mTOR signaling on tumor cell proliferation and apoptosis is well-known in canine OSA, the relation between mTOR and migration, invasion, and angiogenesis properties has been better explored in other types of canine cancer including hemangiosarcoma (194), prostate cancer (195), mammary tumors (196, 197), melanoma (198), and mast cell tumors (199).

Of relevance, MDCK cell model was used to demonstrate that mTOR signaling plays important roles in the regulation of epithelial tubule formation on Matrigel. It was observed that PI3-kinase regulates early epithelial remodeling stages, while mTOR modulates latter stages of tubule development (200), suggesting a possible involvement of mTOR pathway in VM progression. To the best of our knowledge, there are no studies investigating mTOR modulation mediated by the DEPTOR domain in dog.

### RhoA/ROCK in Canine MDCK Cells

Considering that cell migration plays crucial roles in cancer cell invasion, the study of mechanisms of junction and cytoskeletal organization mediated by guanosine triphosphatases (GTPases) of the Rho family has acquired great importance (201, 202). RhoA/ROCK pathway has been widely investigated in MDCK cells as a model of cell migration, cell-cell interaction and adhesion, EMT promotion, and virus entry (201–204).

In Moloney sarcoma virus-(MDCK)-invasive (MSV-MDCK-INV) variant tumor cells, it has been observed that Rho/ROCK activation may affect tumor cell migration and metastasis by stimulating the pseudopodal translocation of mRNAs and thereby regulating the expression of local signaling tumorigenic cascades (205, 206). RhoA hyperactivation can also influence normal MDCK cell polarity (Yu et al., 2008). The inhibition of RhoA pathway leads to a decrease of anchorage-independent growth of MDCK cells *in vitro* and in syngeneic mice, also downregulating *Cox2*gene (207, 208).

## LncRNAs

Non-coding RNAs, especially miRNAs and lncRNAs, have been widely investigated due to their roles as key players in regulating various biological and pathological processes involved in OSA progression, including cancer cell migration, invasion, angiogenesis, and metastasis (209, 210). LncRNAs are non-coding transcripts >than 200 bp in length, and different studies demonstrated the influence of these molecules in gene expression at the epigenetic, transcriptional, and post-transcriptional levels. One of the most classical mechanisms through which lncRNAs regulate gene expression involves their association with chromatin modeling complexes and transcription factors, influencing transcriptional repression and activation of gene promoters (211).

Ren et al. profiled the expression of lncRNAs in highly aggressive OSA cell line 143B in comparison with its parental poorly aggressive cell line HOS, both plated on Matrigel. The top five upregulated lncRNAs were n337322, n333984, n381586, n338209, and TCONS_l2_00028738-XLOC_l2_014777, while the five downregulated lncRNAs were n334144, n342556,n410003, n335665, and ENST00000442174, also indicating that the top-ranked hub lncRNA that had the highest connections with the majority of the others in the network was n340532 (67). Through VM assay, this study also showed that VM ability of 143B cells strongly decreased following n340532 knockdown, as well as the number of metastatic nodules after injection of 143B cells stably transfected with sh-n340532 into nude mice. Tumor tissues collected from the sh-n340532 group exhibited a decreased number of VM channels compared to the control group (67). FTX and MALAT_1_ were also strongly upregulated in this study. As far as FTX is concerned, its involvement in migration and metastasis was also previously demonstrated, as well as the induction of VM by MALAT_1_ (16, 212).

Among others, lncRNA AFAP1-AS1 was found to be aberrantly expressed in OSA together with HOTAIR, HULC, and H19 that were upregulated in human OSA tissues and cell lines. Shi et al. also performed an in-depth investigation to explore the role and the mechanism of AFAP1-AS1 in OSA progression, demonstrating that the stable transfection of different OSA cell lines with siRNA AFAP1-AS1 strongly reduced their ability to form tube-like structures *in vitro*. In the same work, a concomitant decrease of EMT and RhoC/ROCK1/p38MAPK/Twist1 signaling pathway was also observed (213).

Moreover, differences between non-VM and VM cells compared in a microarray highlighted the significant overexpression of the lncRNAs LINC00265 and LINC00342 in the VMOSA cell line with respect to control. The study also confirmed that both LINC00265 and LINC00342 were upregulated in OSA tissues and that the high expression of LINC00265 was positively correlated with Spermine N1-Acetyltransferase 1 (*Sat1*) and Vav Guanine Nucleotide Exchange Factor 3 (*Vav3*) gene expression, as well as with poor prognosis. LINC00265 was also demonstrated to promote proliferation, migration, invasion, and tube formation via miR3825p targeting *Sat1* and *Vav3* genes in OSA cells cultured on Matrigel. SAT1 is a polyamine acetyltransferase that has a controversial role among different tumors, although it has been demonstrated to promote proliferation and metastasis of OSA cells both *in vitro* and *in vivo* (214). VAV3 is an important factor regulating angiogenesis and regulates the Rho/Rac family of GTPases involved in cell growth and motility (214).

### LncRNA in Dogs

Among the multiple epigenetic mechanisms found in canine cancer, DNA methylation and histone modification have been identified on the basis of OSA progression (211). Le Beguec et al. characterized the expression profiles of 10.444 canine lncRNAs in 26 distinct tissue types. Their study showed that lncRNA expression is mainly clustered by tissue type, highlighting that 44% of canine lncRNAs are expressed in a tissue-specific manner and also identifying more than 900 conserved dog-human lncRNAs (215). An alignment-free program that accurately annotates lncRNAs (FEELnc) was used on a real data set of 20 RNA-Seq from 16 different canine tissues, produced by the European LUPA consortium to expand the canine genome annotation, including 10.374 novel lncRNAs and 58.640 mRNAs transcripts (216). This work allowed identifying three new cancer susceptibility candidate lncRNAs in dogs, which are well-described in human cancer, including MALAT_1_, that is associated with human VM and metastasis (16, 217). Other studies observed more than 900 dog-human conserved lncRNAs using comparative genomics, confirming the presence of well-studied lncRNAs in dogs, such as HOTAIR and MALAT_1_ in canine B cell lymphoma and identifying lncRNAs differential expression as a prognostic tool (218–220). Of relevance, 417 differentially expressed lncRNAs were identified in canine oral melanomas in comparison with control samples, including the well-studied lncRNA ZEB2-AS, a lncRNA involved in the regulation of the transcription factor Zinc Finger E-Box Binding Homeobox 2 (*Zeb2*) during EMT in human colon, pancreatic, and breast cancer cell lines, as well as SOX21 Antisense Divergent Transcript 1(*Sox21-as1*) and Cancer Susceptibility 15(*Casc15)* (211, 221, 222). Finally, long non-coding transcripts from telomeres, called telomeric repeat-containing RNA (TERRA), were identified as blocking telomerase activity in canine tumor cell lines originated from soft tissue sarcomas (223). MDCK cells were also tested for the presence of tumorigenic lncRNAs, with the aim of preparing a safer and more reliable non-neoplastic MDCK cell line for vaccine production, founding several tumor-associated lncRNAs (224). Furthermore, a highly upregulated lncRNA in liver cancer was demonstrated to be a promoter during the epithelial and smooth-muscle-like differentiation of adipose-derived stem cells (ADSCs) via the bone morphogenetic protein 9(BMP9)/Wnt/β-catenin/Notch network (225). Genome-wide association studies (GWAS) identified a set of variants within the intron of a lncRNA upstream of the adrenoceptor beta 1(*Adrb1*) gene which is strongly associated with coat color. Two variants were found at high frequency in single-coated dogs and are rare in wolves (226).

## Therapeutic Potential and Current Limitations

Both western and traditional Chinese medicines were used to evaluate a potential VM inhibition. Current anti-angiogenic drugs are often useless in the dampening of VM, inhibiting directly endothelial cell proliferation. At the same time, the consequent vascular density decrease can cause hypoxia in the tissue triggering VM as a compensatory stimulus (2). The combination of drugs targeting VM and classical tumor angiogenesis can definitively reduce the blood and nutrient supply of tumors (227). Furthermore, in the era of chimeric antigen receptor (CAR)-T cell therapy, it is increasingly urgent to find specific markers for cancer management, and VM can represent an opportunity to find a cancer selective therapeutic target. In fact, in the VM process, multipotent tumor cells with CSC-like phenotype can transdifferentiate, generating ECM-rich, CD31-negative, and PAS-positive vascular networks, but CD31+, PAS-negative tubular-like structures have also been observed (6, 59). This evidence demonstrates that the mechanism of endothelial transdifferentiation of cancer cells within the tumor is still unclear, and this issue complicates the identification of specific cancer biomarkers. Recently, increasingly advanced *in vitro* models have been developed for the deeper investigation of this relative new process.

## Conclusion and Perspectives

A growing body of evidence indicates that VM plays fundamental roles in tumor invasion, metastasis, and poor prognosis in human patients with malignant tumors, including OSA. Thus, VM may represent a potential novel target of anti-tumor therapy, even though the cellular mechanisms and molecular pathways by which VM is promoted have not been fully clarified. Endothelial mediators have been especially explored in human OSA and in veterinary oncology, together with the presence of CSC markers and the pathways involved in ECM interaction and cell adhesion. The molecular pathways involving VEGF/VEGFR and integrins have been found to be related to VM and vessel-like formation *in vitro* in canine oncology, while CD133 resulted to be determinant for tubular-like structure formation *in vitro* of canine normal cells (Supplementary Table 1). Information concerning the VM process and its biological implications in cancer is still limited in veterinary literature, despite the importance of canine tumor models in comparative oncology. The current knowledge concerning VM findings in human OSA, summarized in the present review, may provide a basis for stimulating future studies investigating VM in canine oncology as a possible target with great promise in cancer therapy.

## Author Contributions

MM, MR, and LDS conceived and designed the review. MM and MR wrote the review. LDS and RDM supervised and guided the entire project. All authors contributed to the article and approved the submitted version.

## Conflict of Interest

The authors declare that the research was conducted in the absence of any commercial or financial relationships that could be construed as a potential conflict of interest.

## Publisher's Note

All claims expressed in this article are solely those of the authors and do not necessarily represent those of their affiliated organizations, or those of the publisher, the editors and the reviewers. Any product that may be evaluated in this article, or claim that may be made by its manufacturer, is not guaranteed or endorsed by the publisher.
