# Supplementary material for: An Update on Molecular Pathways Regulating Vasculogenic Mimicry in Human Osteosarcoma and Their Role in Canine Oncology
Source: Front Vet Sci. 2021 Sep 23;8:722432. doi: 10.3389/fvets.2021.722432 (PMC8494780; doi:10.3389/fvets.2021.722432)
Supplement: Supplementary file 1 [file Table_1.docx]

**Supplementary Table**

**Table S1.** Molecular pathways related to VM process in human osteosarcoma, their recognized predominant functions and available literature data concerning their evaluations in canine tumor cells and tissues.

| **Molecular pathways of VM in hOSA** | **Main functions** | **Investigations in canine tumour cells and tissues** |
| --- | --- | --- |
| CD133 | **Stem cell marker**; cell motility (1). | OSA(2), glioma (3), melanoma (2), hepatocellular carcinoma (4,5), B-cell lymphoma (6), granular cell tumour (7), insulinoma CSC-like cells (8), prostate cancer cells (9), lung adenocarcinoma (10), hemangiosarcoma (11), transitional cell carcinoma (12), mammary gland adenocarcinoma (13). |
| ALDH1 enzymatic activity | **Stem cell marker**; detoxification of endogenous and exogenous aldehyde substrates; self-renewal, differentiation and self-protection (14). | OSA, haemangiosarcoma, lymphoma, acute lymphoblasticleukaemia (15), melanoma (16), mammary carcinoma (17). |
| VE-cadherin | **Endothelial mediator**; intercellular adhesión (18), vascular homeostasis (19), EMT (20). | OSA (21), mammary tumors (22), thyroid carcinoma, perianal gland epithelioma (23). |
| VEGF/VEGFR | **Endothelial mediator**; vascular homeostasis (24). | OSA (25-30), mammary tumours (31-34), mast cell tumour (35,36), lymphoma (37), perivascular wall tumours (38), hemangiosarcoma (39), skin tumours (40,41), prostate cancer (42). |
| PDGF/PDGFR | **Endothelial mediator**; vascular homeostasis (43). | OSA (44,45–47), astrocytoma (48), fibrosarcoma (49), squamous cell carcinoma (50), lymphoma (51), prostate cancer (52), hemangioma and hemangiosarcoma (53), melanoma (54), mast cell tumors (55), hepatocellular carcinoma (56), mammary tumours (57), nervous system tumors (58,59). |
| FAK | **Response to ECM environment and cell adhesion**; adhesion to the extracellular matrix, cell survival, proliferation and migration (60), EMT (61). | OSA (62), hemangiosarcoma (63), mammary gland tumour (64). |
| Mig7 | **Response to ECM environment and cell adhesion**; adhesion to the extracellular matrix, cell migration (65). | - |
| MMP1, MMP2,  MMP9 | **Response to ECM environment and cell adhesion**; adhesion to the extracellular matrix, cell migration, apoptosis, immunity and angiogenesis (66). | OSA (67–70,30), mast cell tumour (71), lymphoma (72), mammary tumours (73), chondrosarcoma (74), oronasal tumors, hemangiosarcomas, meningiomas (75). |
| Integrins | **Response to ECM environment and cell adhesion**; adhesion to the extracellular matrix, regulation of the actin cytoskeleton, cell adhesion and migration, anchorage-independent growth, EMT (76). | OSA (77), mammary tumors and lymph node metastases (78,79), cutaneous histiocytoma (80), hemangiosarcoma cell lines (81-83) |
| EphA2 | **Response to ECM environment and cell adhesion**; cell motility, angiogenesis (84); virus receptor (85). | OSA (86), high-grade gliomas (87,88), prostate carcinoma cell lines derived from lung and bone metastases (89). |
| mTOR pathway | Cell homeostasis, proliferation and migration (90); HIF-1α stabilizer (91). | OSA (92), hemangiosarcoma (93), prostate cancer (94), mammary tumors (95,96), melanoma (97), mast cell tumors (98). |
| RhoA/ROCK | Cell migration, cell adhesion, EMT and virus entry (99–102). | - |
| lncRNAs | Regulation of various biological and pathological processes. | HOTAIR and MALAT_1_ in B cell lymphoma (103,104), ZEB2-AS, SOX21-AS1 and CASC15 in oral melanomas (105), TERRA in soft tissue sarcoma cell line (106). |

References

1. Jászai J, Thamm K, Karbanová XJ, Janich P, Fargeas CA, Huttner WB, Corbeil D. Prominins control ciliary length throughout the animal kingdom: New lessons from human prominin-1 and zebrafish prominin-3. *J Biol Chem* (2020) **295**:6007–6022. doi:10.1074/jbc.RA119.011253
2. Guth AM, Deogracias M, Dow SW. Comparison of cancer stem cell antigen expression by tumor cell lines and by tumor biopsies from dogs with melanoma and osteosarcoma. *Vet Immunol Immunopathol* (2014) **161**:132–140. doi:10.1016/j.vetimm.2014.07.006
3. Fernández F, Deviers A, Dally C, Mogicato G, Delverdier M, Cauzinille L, Gnirs K, Añor S, de la Fuente C, Fondevila D, et al. Presence of neural progenitors in spontaneous canine gliomas: A histopathological and immunohistochemical study of 20 cases. *Vet J* (2016) **209**:125–132. doi:10.1016/j.tvjl.2015.10.039
4. Fujimoto A, Neo S, Ishizuka C, Kato T, Segawa K, Kawarai S, Ogihara K, Hisasue M, Tsuchiya R. Identification of cell surface antigen expression in canine hepatocellular carcinoma cell lines. *J Vet Med Sci* (2013) **75**:831–835. doi:10.1292/jvms.12-0549
5. Michishita M, Ezaki S, Ogihara K, Naya Y, Azakami D, Nakagawa T, Sasaki N, Arai T, Shida T, Takahashi K. Identification of tumor-initiating cells in a canine hepatocellular carcinoma cell line. *Res Vet Sci* (2014) **96**:315–322. doi:10.1016/j.rvsc.2014.01.004
6. Ito D, Endicott MM, Jubala CM, Helm KM, Burnett RC, Husbands BD, Borgatti A, Henson MS, Burgess KE, Bell JS, et al. A tumor-related lymphoid progenitor population supports hierarchical tumor organization in canine B-cell lymphoma. *J Vet Intern Med* (2011) **25**:890–896. doi:10.1111/j.1939-1676.2011.0756.x
7. Suzuki S, Uchida K, Harada T, Nibe K, Yamashita M, Ono K, Nakayama H. The Origin and Role of Autophagy in the Formation of Cytoplasmic Granules in Canine Lingual Granular Cell Tumors. *Vet Pathol* (2015) **52**:456–464. doi:10.1177/0300985814546051
8. Capodanno Y, Buishand FO, Pang LY, Kirpensteijn J, Mol JA, Argyle DJ. Notch pathway inhibition targets chemoresistant insulinoma cancer stem cells. *Endocr Relat Cancer* (2018) **25**:131–144. doi:10.1530/ERC-17-0415
9. Elshafae SM, Kohart NA, Altstadt LA, Dirksen WP, Rosol TJ. The Effect of a Histone Deacetylase Inhibitor (AR-42) on Canine Prostate Cancer Growth and Metastasis. *Prostate* (2017) **77**:776–793. doi:10.1002/pros.23318
10. Tanabe A, Deguchi T, Sato T, Nemoto Y, Maruo T, Madarame H, Shida T, Naya Y, Ogihara K, Sahara H. Radioresistance of cancer stem-like cell derived from canine tumours. *Vet Comp Oncol* (2016) **14**:e93–e101. doi:10.1111/vco.12110
11. Gorden BH, Kim JH, Sarver AL, Frantz AM, Breen M, Lindblad-Toh K, O’Brien TD, Sharkey LC, Modiano JF, Dickerson EB. Identification of three molecular and functional subtypes in canine hemangiosarcoma through gene expression profiling and progenitor cell characterization. *Am J Pathol* (2014) **184**:985–995. doi:10.1016/j.ajpath.2013.12.025
12. Deguchi T, Hosoya K, Murase Y, Koangyong S, Kim S, Okumura M. Analysis of radiosensitivity of cancer stem-like cells derived from canine cancer cell lines. *Vet Comp Oncol* (2019) **17**:119–129. doi:10.1111/vco.12452
13. Michishita M, Akiyoshi R, Yoshimura H, Katsumoto T, Ichikawa H, Ohkusu-Tsukada K, Nakagawa T, Sasaki N, Takahashi K. Characterization of spheres derived from canine mammary gland adenocarcinoma cell lines. *Res Vet Sci* (2011) **91**:254–260. doi:10.1016/j.rvsc.2010.11.016
14. Tomita H, Tanaka K, Tanaka T, Hara A. Aldehyde dehydrogenase 1A1 in stem cells and cancer. *Oncotarget* (2016) **7**:11018–11032. doi:10.18632/oncotarget.6920
15. Blacking TM, Waterfall M, Samuel K, Argyle DJ. Flow cytometric techniques for detection of candidate cancer stem cell subpopulations in canine tumour models. *Vet Comp Oncol* (2012) **10**:252–273. doi:10.1111/j.1476-5829.2011.00293.x
16. Wilson-Robles HM, Daly M, Pfent C, Sheppard S. Identification and evaluation of putative tumour-initiating cells in canine malignant melanoma cell lines. *Vet Comp Oncol* (2015) **13**:60–69. doi:10.1111/vco.12019
17. Michishita M, Akiyoshi R, Suemizu H, Nakagawa T, Sasaki N, Takemitsu H, Arai T, Takahashi K. Aldehyde dehydrogenase activity in cancer stem cells from canine mammary carcinoma cell lines. *Vet J* (2012) 193:508–513. doi:10.1016/j.tvjl.2012.01.006
18. Heimark RL, Degner M, Schwartz SM. Identification of a Ca2+-dependent cell-cell adhesion molecule in endothelial cells. *J Cell Biol* (1990) **110**:1745–1756. doi:10.1083/jcb.110.5.1745
19. Giannotta M, Trani M, Dejana E. VE-cadherin and endothelial adherens junctions: Active guardians of vascular integrity. *Dev Cell* (2013) **26**:441–454. doi:10.1016/j.devcel.2013.08.020
20. Lu CC, Liu MM, Clinton M, Culshaw G, Argyle DJ, Corcoran BM. Developmental pathways and endothelial to mesenchymal transition in canine myxomatous mitral valve disease. *Vet J* (2015)
21. Izumi Y, Takagi S. Vascular disrupting effect of combretastatin A-4 phosphate with inhibition of vascular endothelial cadherin in canine osteosarcoma-xenografted mice. *Res Vet Sci* (2019) **122**:1–6. doi:10.1016/j.rvsc.2018.10.017
22. Nam AR, Lee KH, Hwang HJ, Schabort JJ, An JH, Won SH, Cho JY. Alternative methylation of intron motifs is associated with cancer-related gene expression in both canine mammary tumor and human breast cancer. *Clin Epigenetics* (2020) **12**: doi:10.1186/s13148-020-00888-4
23. Izumi Y, Aoshima K, Hoshino Y, Takagi S. Effects of combretastatin A-4 phosphate on canine normal and tumor tissue-derived endothelial cells. *Res Vet Sci* (2017) **112**:222–228. doi:10.1016/j.rvsc.2017.05.017
24. Karaman S, Leppänen VM, Alitalo K. Vascular endothelial growth factor signaling in development and disease. *Dev* (2018) **145**: doi:10.1242/dev.151019
25. Tanabe A, Kobayashi D, Maeda K, Taguchi M, Sahara H. Angiogenesis-related gene expression profile in clinical cases of canine cancer. *Vet Med Sci* (2019) **5**:19–29. doi:10.1002/vms3.127
26. Cam M, Gardner HL, Roberts RD, Fenger JM, Guttridge DC, London CA, Cam H. ΔNp63 mediates cellular survival and metastasis in canine osteosarcoma. *Oncotarget* (2016) **7**:48533–48546. doi:10.18632/oncotarget.10406
27. Lopez CM, Yu PY, Zhang X, Yilmaz AS, London CA, Fenger JM. MiR-34a regulates the invasive capacity of canine osteosarcoma cell lines. *PLoS One* (2018) **13**: doi:10.1371/journal.pone.0190086
28. Sánchez-Céspedes R, Accornero P, Miretti S, Martignani E, Gattino F, Maniscalco L, Gola C, Iussich S, Martano M, Morello E, et al. In vitro and in vivo effects of toceranib phosphate on canine osteosarcoma cell lines and xenograft orthotopic models. *Vet Comp Oncol* (2020) **18**:117–127. doi:10.1111/vco.12562
29. Massimini M, De Maria R, Malatesta D, Romanucci M, D’Anselmo A, Della Salda L. Establishment of three-dimensional canine osteosarcoma cell lines showing vasculogenic mimicry and evaluation of biological properties after treatment with 17-AAG. *Vet Comp Oncol* (2019) **17**:376–384. doi:10.1111/vco.12482
30. Fossey SL, Bear MD, Kisseberth WC, Pennell M, London CA. Oncostatin M promotes STAT3 activation, VEGF production, and invasion in osteosarcoma cell lines. *BMC Cancer* (2011) **11**:1–10. doi:10.1186/1471-2407-11-125
31. Prado MCM, Macedo S de AL, Guiraldelli GG, de Faria Lainetti P, Leis-Filho AF, Kobayashi PE, Laufer-Amorim R, Fonseca-Alves CE. Investigation of the Prognostic Significance of Vasculogenic Mimicry and Its Inhibition by Sorafenib in Canine Mammary Gland Tumors. *Front Oncol* (2019) **9**: doi:10.3389/fonc.2019.01445
32. Queiroga FL, Pires I, Parente M, Gregório H, Lopes CS. COX-2 over-expression correlates with VEGF and tumour angiogenesis in canine mammary cancer. *Vet J* (2011) **189**:77–82. doi:10.1016/j.tvjl.2010.06.022
33. Qiu C, Lin DD, Wang HH, Qiao CH, Wang J, Zhang T. Quantification of VEGF-C expression in canine mammary tumours. *Aust Vet J* (2008) **86**:279–282. doi:10.1111/j.1751-0813.2008.00312.x
34. Santos A, Lopes C, Gärtner F, Matos AJF. VEGFR-2 expression in malignant tumours of the canine mammary gland: a prospective survival study. *Vet Comp Oncol* (2016) **14**:e83–e92. doi:10.1111/vco.12107
35. Rebuzzi L, Willmann M, Sonneck K, Gleixner K V., Florian S, Kondo R, Mayerhofer M, Vales A, Gruze A, Pickl WF, et al. Detection of vascular endothelial growth factor (VEGF) and VEGF receptors Flt-1 and KDR in canine mastocytoma cells. *Vet Immunol Immunopathol* (2007) **115**:320–333. doi:10.1016/j.vetimm.2006.11.009
36. Thompson JJ, Morrison JA, Pearl DL, Boston SE, Wood GA, Foster RA, Coomber BL. Receptor Tyrosine Kinase Expression Profiles in Canine Cutaneous and Subcutaneous Mast Cell Tumors. *Vet Pathol* (2016) **53**:545–558. doi:10.1177/0300985815610388
37. Wolfesberger B, Tonar Z, Fuchs-Baumgartinger A, Walter I, Skalicky M, Witter K, Thalhammer JG, Pagitz M, Kleiter M. Angiogenic markers in canine lymphoma tissues do not predict survival times in chemotherapy treated dogs. *Res Vet Sci* (2012) **92**:444–450. doi:10.1016/j.rvsc.2011.04.018
38. Avallone G, Stefanello D, Boracchi P, Ferrari R, Gelain ME, Turin L, Tresoldi E, Roccabianca P. Growth Factors and COX2 Expression in Canine Perivascular Wall Tumors. *Vet Pathol* (2015) **52**:1034–1040. doi:10.1177/0300985815575050
39. Kodama A, Sakai H, Matsuura S, Murakami M, Murai A, Mori T, Maruo K, Kimura T, Masegi T, Yanai T. Establishment of canine hemangiosarcoma xenograft models expressing endothelial growth factors, their receptors, and angiogenesis-associated homeobox genes. *BMC Cancer* (2009) **9**:363. doi:10.1186/1471-2407-9-363
40. Al-Dissi AN, Haines DM, Singh B, Kidney BA. Immunohistochemical expression of vascular endothelial growth factor and vascular endothelial growth factor receptor associated with tumor cell proliferation in canine cutaneous squamous cell carcinomas and trichoepitheliomas. *Vet Pathol* (2007) **44**:823–830. doi:10.1354/vp.44-6-823
41. Rawlings NG, Simko E, Bebchuk T, Caldwell SJ, Singh B. Localization of integrin αvβ3 and vascular endothelial growth factor receptor-2 (KDR/Flk-1) in cutaneous and oral melanomas of dog. *Histol Histopathol* (2003) **18**:819–826. doi:10.14670/HH-18.819
42. Chevalier S, Defoy I, Lacoste J, Hamel L, Guy L, Bégin LR, Aprikian AG. Vascular endothelial growth factor and signaling in the prostate: more than angiogenesis. *Mol Cell Endocrinol* (2002) **189**:169–179. doi:10.1016/S0303-7207(01)00728-6
43. Kazlauskas A. PDGFs and their receptors. *Gene* (2017) **614**:1–7. doi:10.1016/j.gene.2017.03.003
44. Maniscalco L, Iussich S, Morello E, Martano M, Biolatti B, Riondato F, Salda L Della, Romanucci M, Malatesta D, Bongiovanni L, et al. PDGFs and PDGFRs in canine osteosarcoma: New targets for innovative therapeutic strategies in comparative oncology. *Vet J* (2013) **195**:41–47. doi:10.1016/j.tvjl.2012.05.003
45. Gentilini F, Capitani O, Tinto D, Rigillo A, Sabattini S, Bettini G, Turba Maria E. Assessment of PDGFRβ promoter methylation in canine osteosarcoma using methylation-sensitive high-resolution melting analysis. *Vet Comp Oncol* (2020) **18**:484–493. doi:10.1111/vco.12567
46. Sánchez-Céspedes R, Accornero P, Miretti S, Martignani E, Gattino F, Maniscalco L, Gola C, Iussich S, Martano M, Morello E, et al. In vitro and in vivo effects of toceranib phosphate on canine osteosarcoma cell lines and xenograft orthotopic models. *Vet Comp Oncol* (2020) **18**:117–127. doi:10.1111/vco.12562
47. Alegre F, Ormonde AR, Snider KM, Woolard K, Yu AM, Wittenburg LA. A genetically engineered microRNA-34a prodrug demonstrates anti-tumor activity in a canine model of osteosarcoma. *PLoS One* (2018) **13**: doi:10.1371/journal.pone.0209941
48. Higgins RJ, Dickinson PJ, Lecouteur RA, Bollen AW, Wang H, Wang H, Corely LJ, Moore LM, Zang W, Fuller GN. Spontaneous canine gliomas: Overexpression of EGFR, PDGFRα and IGFBP2 demonstrated by tissue microarray immunophenotyping. *J Neurooncol* (2010) **98**:49–55. doi:10.1007/s11060-009-0072-5
49. Ahmed I, Sozmen M. Expression of PDGF-A, PDGFRA, integrin subunit alpha V and selectin E is increased in canine cutaneous fibrosarcomas. *Biotech Histochem* (2020) **9**;19.doi:10.1080/10520295.2020.1832256
50. Sözmen M, Devrim AK, Sudağıdan M, Kabak YB, Yıldırım F. Expression of angiogenic growth factors in canine squamous cell cancers. *Biotech Histochem* (2020) doi:10.1080/10520295.2020.1818826
51. Holtermann N, Kiupel M, Kessler M, Teske E, Betz D, Hirschberger J. Masitinib monotherapy in canine epitheliotropic lymphoma. *Vet Comp Oncol* (2016) **14**:127–135. doi:10.1111/vco.12157
52. Kobayashi PE, Lainetti PF, Leis-Filho AF, Delella FK, Carvalho M, Cury SS, Carvalho RF, Fonseca-Alves CE, Laufer-Amorim R. Transcriptome of Two Canine Prostate Cancer Cells Treated With Toceranib Phosphate Reveals Distinct Antitumor Profiles Associated With the PDGFR Pathway. *Front Vet Sci* (2020) **7**: doi:10.3389/fvets.2020.561212
53. Abou Asa S, Murai A, Murakami M, Hoshino Y, Mori T, Maruo K, Khater A, El-Sawak A, Abd El-Aziz E, Yanai T, et al. Expression of platelet-derived growth factor and its receptors in spontaneous canine hemangiosarcoma and cutaneous hemangioma. *Histol Histopathol* (2012) **27**:601–607. doi:10.14670/HH-27.601
54. Iussich S, Maniscalco L, Di Sciuva A, Iotti B, Morello E, Martano M, Gattino F, Buracco P, De Maria R. PDGFRs expression in dogs affected by malignant oral melanomas: correlation with prognosis. *Vet Comp Oncol* (2017) **15**:462–469. doi:10.1111/vco.12190
55. Takeuchi Y, Fujino Y, Watanabe M, Nakagawa T, Ohno K, Sasaki N, Sugano S, Tsujimoto H. Screening of therapeutic targets for canine mast cell tumors from a variety of kinase molecules. *J Vet Med Sci* (2011) **73**:1295–1302. doi:10.1292/jvms.11-0093
56. Iida G, Asano K, Seki M, Sakai M, Kutara K, Ishigaki K, Kagawa Y, Yoshida O, Teshima K, Edamura K, et al. Gene expression of growth factors and growth factor receptors for potential targeted therapy of canine hepatocellular carcinoma. *J Vet Med Sci* (2014) **76**:301–306. doi:10.1292/jvms.13-0378
57. Li W, Guo M, Liu Y, Mu W, Deng G, Li C, Qiu C. Selenium Induces an Anti-tumor Effect Via Inhibiting Intratumoral Angiogenesis in a Mouse Model of Transplanted Canine Mammary Tumor Cells. *Biol Trace Elem Res* (2016) **171**:371–379. doi:10.1007/s12011-015-0554-6
58. Whelan HT, Przybylski C, Bajic DM, Schmidt MH. Intracellular growth factor metabolism in proliferation of a brain tumor cell line - Intracellular growth factors and brain tumor proliferation. *J Neurooncol* (1993) **15**:243–250. doi:10.1007/BF01050070
59. Muscatello LV, Avallone G, Serra F, Seuberlich T, Mandara MT, Sisó S, Brunetti B, Oevermann A. Glomeruloid Microvascular Proliferation, Desmoplasia, and High Proliferative Index as Potential Indicators of High Grade Canine Choroid Plexus Tumors. *Vet Pathol* (2018) **55**:391–401. doi:10.1177/0300985817754124
60. Lv PC, Jiang AQ, Zhang WM, Zhu HL. FAK inhibitors in Cancer, a patent review. *Expert Opin Ther Pat* (2018) 28:139–145. doi:10.1080/13543776.2018.1414183
61. Selvaggio G, Canato S, Pawar A, Monteiro PT, Guerreiro PS, Brás MM, Janody F, Chaouiya C. Hybrid Epithelial-Mesenchymal Phenotypes Are Controlled by Microenvironmental Factors. *Cancer Res* (2020) **80**:2407–2420. doi:10.1158/0008-5472.CAN-19-3147
62. Rizzo VL, Levine CB, Wakshlag JJ. The effects of sulforaphane on canine osteosarcoma proliferation and invasion. *Vet Comp Oncol* (2017) **15**:718–730. doi:10.1111/vco.12212
63. Marley K, Maier CS, Helfand SC. Phosphotyrosine enrichment identifies focal adhesion kinase and other tyrosine kinases for targeting in canine hemangiosarcoma. *Vet Comp Oncol* (2012) 10:214–222. doi:10.1111/j.1476-5829.2012.00325.x
64. Lee JL, Chang CJ, Chueh LL, Lin CT. Secreted Frizzled Related Protein 2 (sFRP2) decreases susceptibility to UV-induced apoptosis in primary culture of canine mammary gland tumors by NF-κB activation or JNK suppression. *Breast Cancer Res Treat* (2006) **100**:49–58. doi:10.1007/s10549-006-9233-9
65. Robertson GP. Mig-7 linked to vasculogenic mimicry. *Am J Pathol* (2007) **170**:1454–1456. doi:10.2353/ajpath.2007.070127
66. Gobin E, Bagwell K, Wagner J, Mysona D, Sandirasegarane S, Smith N, Bai S, Sharma A, Schleifer R, She JX. A pan-cancer perspective of matrix metalloproteases (MMP) gene expression profile and their diagnostic/prognostic potential. *BMC Cancer* (2019) **19**: doi:10.1186/s12885-019-5768-0
67. Hadjimichael AC, Foukas AF, Savvidou OD, Mavrogenis AF, Psyrri AK, Papagelopoulos PJ. The anti-neoplastic effect of doxycycline in osteosarcoma as a metalloproteinase (MMP) inhibitor: a systematic review. *Clin Sarcoma Res* (2020) **10**: doi:10.1186/s13569-020-00128-6
68. Lana SE, Ogilvie GK, Hansen RA, Powers BE, Dernell WS, Withrow SJ. Identification of matrix metalloproteinases in canine neoplastic tissue. *Am J Vet Res* (2000) **61**:111–114. doi:10.2460/ajvr.2000.61.111
69. Nytko KJ, Thumser-Henner P, Russo G, Weyland MS, Rohrer Bley C. Role of HSP70 in response to (thermo)radiotherapy: analysis of gene expression in canine osteosarcoma cells by RNA-seq. *Sci Rep* (2020) **10**: doi:10.1038/s41598-020-69619-2
70. Loukopoulos P, O’Brien T, Ghoddusi M, Mungall BA, Robinson WF. Characterisation of three novel canine osteosarcoma cell lines producing high levels of matrix metalloproteinases. *Res Vet Sci* (2004) **77**:131–141. doi:10.1016/j.rvsc.2004.01.006
71. Giantin M, Aresu L, Benali S, Aricò A, Morello EM, Martano M, Vascellari M, Castagnaro M, Lopparelli RM, Zancanella V, et al. Expression of Matrix Metalloproteinases, Tissue Inhibitors of Metalloproteinases and Vascular Endothelial Growth Factor in Canine Mast Cell Tumours. *J Comp Pathol* (2012) **147**:419–429. doi:10.1016/j.jcpa.2012.01.011
72. Gentilini F, Calzolari C, Turba ME, Agnoli C, Fava D, Forni M, Bergamini PF. Prognostic value of serum vascular endothelial growth factor (VEGF) and plasma activity of matrix metalloproteinase (MMP) 2 and 9 in lymphoma-affected dogs. *Leuk Res* (2005) **29**:1263–1269. doi:10.1016/j.leukres.2005.04.005
73. Raposo TP, Beirão BCB, Pires I, Prada J, Brilhante P, Argyle DJ, Queiroga FL. Immunohistochemical expression of CCR2, CSF1R and MMP9 in canine inflammatory mammary carcinomas. Anticancer Res (2016) 36:1805–1813. Available at: https://pubmed.ncbi.nlm.nih.gov/27069163/ [Accessed August 5, 2021]
74. Aresu L, Giantin M, Morello E, Vascellari M, Castagnaro M, Lopparelli R, Zancanella V, Granato A, Garbisa S, Aricò A, et al. Matrix metalloproteinases and their inhibitors in canine mammary tumors. *BMC Vet Res* (2011) **7**:33. doi:10.1186/1746-6148-7-33
75. Pulz LH, Strefezzi RF. Proteases as prognostic markers in human and canine cancers. *Vet Comp Oncol* (2017) 15:669–683. doi:10.1111/vco.12223
76. Pan B, Guo J, Liao Q, Zhao Y. β1 and β3 integrins in breast, prostate and pancreatic cancer: A novel implication (Review). *Oncol Lett* (2018) **15**:5412–5416. doi:10.3892/ol.2018.8076
77. Selvarajah GT, Kirpensteijn J, van Wolferen ME, Rao NAS, Fieten H, Mol JA. Gene expression profiling of canine osteosarcoma reveals genes associated with short and long survival times. *Mol Cancer* (2009) **8**:72. doi:10.1186/1476-4598-8-72
78. Sánchez-Céspedes R, Millán Y, Guil-Luna S, Reymundo C, Espinosa de los Monteros A, Martín de las Mulas J. Myoepithelial cells in canine mammary tumours. *Vet J* (2016) **207**:45–52. doi:10.1016/j.tvjl.2015.10.035
79. Peña L, Nieto A, Perez Alenza MD, Rodriguez A, Sanchez MA, Castaño M. Expression of fibronectin and its integrin receptor α5β1 in canine mammary tumours. *Res Vet Sci* (1994) **57**:358–364. doi:10.1016/0034-5288(94)90131-7
80. Moore PF, Schrenzel MD, Affolter VK, Olivry T, Naydan D. Canine cutaneous histiocytoma is an epidermotropic langerhans cell histiocytosis that expresses CD1 and specific β2-integrin molecules. *Am J Pathol* (1996) **148**:1699–1708
81. Akhtar N, Padilla ML, Dickerson EB, Steinberg H, Breen M, Auerbach R, Helfand SC. Interleukin-12 Inhibits Tumor Growth in a Novel Angiogenesis Canine Hemangiosarcoma Xenograft Model. *Neoplasia* (2004) **6**:106–116. doi:10.1593/neo.03334
82. Fosmire SP, Dickerson EB, Scott AM, Bianco SR, Pettengill MJ, Meylemans H, Padilla M, Frazer-Abel AA, Akhtar N, Getzy DM, et al. Canine malignant hemangiosarcoma as a model of primitive angiogenic endothelium. *Lab Investig* (2004) **84**:562–572. doi:10.1038/labinvest.3700080
83. Olivry T, Marsella R, Hillier A. The ACVD task force on canine atopic dermatitis (XXIII): Are essential fatty acids effective? *Vet Immunol Immunopathol* (2001) **81**:347–362. doi:10.1016/S0165-2427(01)00316-6
84. Ieguchi K, Maru Y. Roles of EphA1/A2 and ephrin-A1 in cancer. *Cancer Sci* (2019) **110**:841–848. doi:10.1111/cas.13942
85. Zhang H, Li Y, Wang HB, Zhang A, Chen ML, Fang ZX, Dong XD, Li SB, Du Y, Xiong D, et al. Ephrin receptor A2 is an epithelial cell receptor for Epstein-Barr virus entry. *Nat Microbiol* (2018) **3**:164–171. doi:10.1038/s41564-017-0080-8
86. Laut MM, Akter SH, Irac SE, Palmieri C. Immunohistochemical expression of EphA2 and EphA3 receptors in canine osteosarcoma. *J Comp Pathol* (2019) **166**:124. doi:10.1016/j.jcpa.2018.10.075
87. Ito K, Miyamoto R, Tani H, Kurita S, Kobayashi M, Tamura K, Bonkobara M. Effect of dasatinib in a xenograft mouse model of canine histiocytic sarcoma and in vitro expression status of its potential target EPHA2. *J Vet Pharmacol Ther* (2018) **41**:e45–e48. doi:10.1111/jvp.12449
88. Kamoun WS, Kirpotin DB, Huang ZR, Tipparaju SK, Noble CO, Hayes ME, Luus L, Koshkaryev A, Kim J, Olivier K, et al. Antitumour activity and tolerability of an EphA2-targeted nanotherapeutic in multiple mouse models. *Nat Biomed Eng* (2019) **3**:264–280. doi:10.1038/s41551-019-0385-4
89. Walker-Daniels J, Coffman K, Azimi M, Rhim JS, Bostwick DG, Snyder P, Kerns BJ, Waters DJ, Kinch MS. Overexpression of the EphA2 tyrosine kinase in prostate cancer. *Prostate* (1999) **41**:275–280. doi:10.1002/(SICI)1097-0045(19991201)41:4<275::AID-PROS8>3.0.CO;2-T
90. Liu J, Wada Y, Katsura M, Tozawa H, Erwin N, Kapron CM, Bao G, Liu J. Rho-associated coiled-coil kinase (ROCK) in molecular regulation of angiogenesis. *Theranostics* (2018) **8**:6053–6069. doi:10.7150/thno.30305
91. Zhang JG, Zhou HM, Zhang X, Mu W, Hu JN, Liu GL, Li Q. Hypoxic induction of vasculogenic mimicry in hepatocellular carcinoma: Role of HIF-1 α, RhoA/ROCK and Rac1/PAK signaling. *BMC Cancer*
92. Gordon IK, Ye F, Kent MS. Evaluation of the mammalian target of rapamycin pathway and the effect of rapamycin on target expression and cellular proliferation in osteosarcoma cells from dogs. *Am J Vet Res* (2008) **69**:1079–1084. doi:10.2460/ajvr.69.8.1079
93. Pyuen AA, Meuten T, Rose BJ, Thamm DH. In vitro effects of PI3K/mTOR inhibition in canine hemangiosarcoma. *PLoS One* (2018) **13**: doi:10.1371/journal.pone.0200634
94. Rivera-Calderón LG, Fonseca-Alves CE, Kobayashi PE, Carvalho M, Vasconcelos RO, Laufer-Amorim R. p-mTOR, p-4EBP-1 and eIF4E expression in canine prostatic carcinoma. *Res Vet Sci* (2019) **122**:86–92. doi:10.1016/j.rvsc.2018.11.006
95. Kim JW, Mahiddine FY, Kim GA. Leptin modulates the metastasis of canine inflammatory mammary adenocarcinoma cells through downregulation of lysosomal protective protein cathepsin a (Ctsa). *Int J Mol Sci* (2020) **21**:1–14. doi:10.3390/ijms21238963
96. Zhao Y, Lin Z, Lin Z, Zhou C, Liu G, Lin J, Zhang D, Lin D. Overexpression of mucin 1 suppresses the therapeutical efficacy of disulfiram against canine mammary tumor. *Animals* (2021) **11**:1–13. doi:10.3390/ani11010037
97. Wei BR, Hoover SB, Peer CJ, Dwyer JE, Adissu HA, Shankarappa P, Yang H, Lee M, Peat TJ, Figg WD, et al. Efficacy, tolerability, and pharmacokinetics of combined targeted MEK and dual mTORC1/2 inhibition in a preclinical model of mucosal melanoma. *Mol Cancer Ther* (2020) **19**:2308–2318. doi:10.1158/1535-7163.MCT-19-0858
98. Rebuzzi L, Willmann M, Sonneck K, Gleixner K V., Florian S, Kondo R, Mayerhofer M, Vales A, Gruze A, Pickl WF, et al. Detection of vascular endothelial growth factor (VEGF) and VEGF receptors Flt-1 and KDR in canine mastocytoma cells. *Vet Immunol Immunopathol* (2007) **115**:320–333. doi:10.1016/j.vetimm.2006.11.009
99. Isozaki Y, Sakai K, Kohiro K, Kagoshima K, Iwamura Y, Sato H, Rindner D, Fujiwara S, Yamashita K, Mizuno K, et al. The Rho-guanine nucleotide exchange factor Solo decelerates collective cell migration by modulating the Rho-ROCK pathway and keratin networks. *Mol Biol Cell* (2020) **31**:741–752. doi:10.1091/MBC.E19-07-0357
100. Citi S, Guerrera D, Spadaro D, Shah J. Epithelial junctions and Rho family GTPases: The zonular signalosome. *Small GTPases* (2014) **5**: doi:10.4161/21541248.2014.973760
101. Fernández-Muñoz B, Yurrita MM, Martín-Villar E, Carrasco-Ramírez P, Megías D, Renart J, Quintanilla M. The transmembrane domain of podoplanin is required for its association with lipid rafts and the induction of epithelial-mesenchymal transition. *Int J Biochem Cell Biol* (2011) **43**:886–896. doi:10.1016/j.biocel.2011.02.010
102. Soliman M, Cho EH, Park JG, Kim JY, Alfajaro MM, Baek Y Bin, Kim DS, Kang M Il, Park SI, Cho KO. Rotavirus-Induced Early Activation of the RhoA/ROCK/MLC Signaling Pathway Mediates the Disruption of Tight Junctions in Polarized MDCK Cells. *Sci Rep* (2018) **8**: doi:10.1038/s41598-018-32352-y
103. Verma A, Jiang Y, Du W, Fairchild L, Melnick A, Elemento O. Transcriptome sequencing reveals thousands of novel long non-coding RNAs in B cell lymphoma. *Genome Med* (2015) **7**:110. doi:10.1186/s13073-015-0230-7
104. Cascione L, Giudice L, Ferraresso S, Marconato L, Giannuzzi D, Napoli S, Bertoni F, Giugno R, Aresu L. Long non-coding RNAs as molecular signatures for canine B-cell lymphoma characterization. *Non-coding RNA* (2019) **5**: doi:10.3390/ncrna5030047
105. Hitte C, Le Béguec C, Cadieu E, Wucher V, Primot A, Prouteau A, Botherel N, Hédan B, Lindblad-Toh K, André C, et al. Genome-wide analysis of long non-coding RNA profiles in canine oral melanomas. *Genes (Basel)* (2019) **10**: doi:10.3390/genes10060477
106. Kreilmeier T, Mejri D, Hauck M, Kleiter M, Holzmann K. Telomere transcripts target telomerase in human cancer cells. *Genes (Basel)* (2016) **7**: doi:10.3390/genes7080046
